# The DNA Helicase Recql4 Is Required for Normal Osteoblast Expansion and Osteosarcoma Formation

**DOI:** 10.1371/journal.pgen.1005160

**Published:** 2015-04-10

**Authors:** Alvin J. M. Ng, Mannu K. Walia, Monique F. Smeets, Anthony J. Mutsaers, Natalie A. Sims, Louise E. Purton, Nicole C. Walsh, T. John Martin, Carl R. Walkley

**Affiliations:** 1 St. Vincent’s Institute of Medical Research, Fitzroy, Victoria, Australia; 2 Department of Medicine, St. Vincent’s Hospital, The University of Melbourne, Fitzroy, Victoria, Australia; 3 ACRF Rational Drug Discovery Centre, St. Vincent’s Institute of Medical Research, Fitzroy, Victoria, Australia; Vanderbilt University, United States of America

## Abstract

*RECQL4* mutations are associated with Rothmund Thomson Syndrome (RTS), RAPADILINO Syndrome and Baller-Gerold Syndrome. These patients display a range of benign skeletal abnormalities such as low bone mass. In addition, RTS patients have a highly increased incidence of osteosarcoma (OS). The role of RECQL4 in normal adult bone development and homeostasis is largely uncharacterized and how mutation of *RECQL4* contributes to OS susceptibility is not known. We hypothesised that Recql4 was required for normal skeletal development and both benign and malignant osteoblast function, which we have tested in the mouse. *Recql4* deletion *in vivo* at the osteoblastic progenitor stage of differentiation resulted in mice with shorter bones and reduced bone volume, assessed at 9 weeks of age. This was associated with an osteoblast intrinsic decrease in mineral apposition rate and bone formation rate in the *Recql4*-deficient cohorts. Deletion of *Recql4* in mature osteoblasts/osteocytes *in vivo*, however, did not cause a detectable phenotype. Acute deletion of *Recql4* in primary osteoblasts or shRNA knockdown in an osteoblastic cell line caused failed proliferation, accompanied by cell cycle arrest, induction of apoptosis and impaired differentiation. When cohorts of animals were aged long term, the loss of *Recql4* alone was not sufficient to initiate OS. We then crossed the Recql4^fl/fl^ allele to a fully penetrant OS model (*Osx*-Cre *p53^fl/fl^*). Unexpectedly, the *Osx*-Cre *p53^fl/fl^Recql4^fl/fl^* (dKO) animals had a significantly increased OS-free survival compared to *Osx*-Cre *p53^fl/fl^* or *Osx*-Cre *p53^fl/fl^Recql4^fl/+^* (het) animals. The extended survival was explained when the Recql4 status in the tumors that arose was assessed, and in no case was there complete deletion of *Recql4* in the dKO OS. These data provide a mechanism for the benign skeletal phenotypes of *RECQL4* mutation syndromes. We propose that tumor suppression and osteosarcoma susceptibility are most likely a function of mutant, not null, alleles of *RECQL4*.

## Author Summary

Rothmund Thomson Syndrome (RTS), RAPADILINO Syndrome and Baller-Gerold Syndrome are very rare human syndromes associated with mutations in RECQL4. RECQL4 is important for controlling how cells divide and for preventing genome damage. Patients with RECQL4 mutations have problems with bone formation and a low bone mass, similar to osteoporosis. RTS patients have a highly increased risk of developing bone cancer (osteosarcoma). The role of RECQL4 in normal bone development and osteosarcoma formation is largely unknown. We have used mouse models to understand the specific role of Recql4 in bone development. Mice with Recql4 removed specifically from their bone cells have shortened bones and a reduced rate of bone formation. Therefore, RECQL4 is essential for normal bone development. Interestingly, the animals with no Recql4 in bone cells did not develop osteosarcoma. Using mouse models of osteosarcoma, we observed delayed cancer formation when Recql4 was also deleted. Further analysis demonstrated that bone cancer could not arise from *Recql4* null cells even with concurrent *p53* deletion. These studies clarify the role of RECQL4 in both normal and malignant bone biology and suggest that RECQL4 mutations that cause osteosarcoma most likely result in proteins with reduced, but not absent, function.

## Introduction

Rothmund-Thomson syndrome (RTS), RAPADILINO Syndrome and Baller-Gerold Syndrome are rare autosomal recessive disorders that are associated with mutations in the DNA helicase *RECQL4*. RECQL4 belongs to a family of RecQ helicases that are important in the regulation of DNA repair and replication, and constitutes five human members: *BLM*, *WRN*, *RECQL4*, *RECQ1 and RECQ5* [1]. Along with *RECQL4*, *WRN* and *BLM* are also associated with human hereditary disorders involving both skeletal defects and cancer predisposition [1]. Mutations in *WRN* cause Werner Syndrome, in *BLM* Bloom Syndrome and when *RECQL4* is mutated RTS and related syndromes arise [1,2]. Abnormalities in skeletal development are a defining feature of the human disorders associated with mutations in RecQ helicases, such as short stature, osteoporosis, low bone mass and polydactyly [3–6]. In addition to the developmental phenotypes shared across RecQ helicase mutation kindreds, RTS patients are predisposed to develop osteosarcoma (OS) [6,7]. However, neither the role of *RECQL4* in osteoblast biology nor the influence of *RECQL4* mutations in the initiation and maintenance of OS have been defined.

OS is the most common primary tumor of bone and it is currently treated with chemotherapy and surgical resection [8–10]. It presents bi-modally, primarily in children and teenagers with a second incidence after the age of 70 [11]. Recent data has begun to shed light on the complex genetics of conventional OS [12], and have revealed that OS is characterised by multiple somatic mutations and chromosomal aberrations [13]. Strikingly, with the exception of *TP53*, there are few highly recurrent somatic alterations [12]. These data highlight that OS is a disease most broadly characterised by a tolerance of genomic instability and mutation burden. Whilst these studies have begun to provide information about the landscape of the OS genome, the initiating events that enable OS formation are less clear. Conversely, human familial cancer syndromes have provided great insight into the genetic initiating events of OS.

Three familial syndromes are strongly associated with a predisposition to OS: Li-Fraumeni syndrome (*TP53* mutations), hereditary retinoblastoma (*RB1* mutations) and RTS. Indeed, mutations in the *TP53* occur in >90% of conventional OS, and this observation is supported by murine OS models that demonstrate a *p53* pathway mutation dependence [12,14–18]. In contrast to both *TP53* and *RB1* pathways, mutations in *RECQL4* have not been observed in sporadic OS. Indeed, elevated levels of *RECQL4* have been reported in sporadic OS, a result potentially confounded by the close genomic linkage of the *RECQL4* locus with *MYC*, which is amplified in many OS [19,20]. Despite the apparently contradictory nature of the data, about 30% of RTS patients with deleterious *RECQL4* mutations develop OS [7]. RTS associated mutations in *RECLQ4* are notable in that they display an unusually high proportion of mutations that impact on splicing, possibly resulting from the presence of numerous short introns, and that mutations spare the N-termial region of protein [21–23]. A primary function of RECQL4 is thought to be mediated by its ATP-dependent helicase RecQ domain, and RTS associated mutations cluster to this region [21]. These clinical observations raise several important questions about the role of *RECQL4* in osteoblast biology and tumorigenesis: firstly, what is the function of *RECQL4* that leads to the benign skeletal defects observed in RTS patients? Secondly, what role does *RECQL4* play in OS initiation and maintenance?

Previous attempts to generate *Recql4* deficient mice have yielded three non-conditional alleles with divergent phenotypes. The first reported allele deleted exons 5–8, resulting in early embryonic lethality [24]. A second allele, generated by an in-frame deletion of exon 13, encoding part of the RecQ helicase domain, resulted in 95% of the mice dying within two weeks of birth [25]. The small proportions of viable homozygous mutants displayed severe growth retardation, but were not reported to develop OS. The third allele replaced part of exon 9 through to 13 with a PGK-Hprt mini gene cassette [26]. Although most homozygous mutant mice were viable, a small proportion displayed skeletal defects such as polydactyly and shorter limbs. These mice also developed cancers including OS, at low penetrance (5% incidence of cancer overall, 40% of cancers were OS (n = 2 animals)). The latter two alleles were both characterised by the presence of aberrant *Recql4* transcripts within the cells, which potentially resulted in the generation of C-terminal truncated proteins. These limitations confound the understanding of the *in vivo* role of *Recql4* in both normal development and tumor formation.

We recently described a conditional murine *Recql4* allele generated by flanking exons 9 and 10 with loxP elements [27]. This region was selected to enable inactivation of the helicase domain and reflect the preponderance of mutations in this region in humans with RTS. In the mouse, exon 9 encodes the start of the ATP dependent RecQ helicase domain. Germ-line deletion of exons 9 and 10 resulted in embryonic lethality prior to E10.5, most similar to the phenotype reported with deletions of exons 5–8. Somatic deletion of *Recql4* in adult mice resulted in the rapid development of a fully penetrant bone marrow failure-like syndrome and death of the *Recql4* deficient animals [27]. This model precluded analysis of the role of *Recql4* in skeletal development and OS. We have now used targeting deletion of this conditional allele within the osteoblast lineage to understand the requirement for *Recql4* in normal skeletal homeostasis and OS development.

## Results

### Generation of osteoblast specific *Recql4* deficient mice

**Fig 1:**
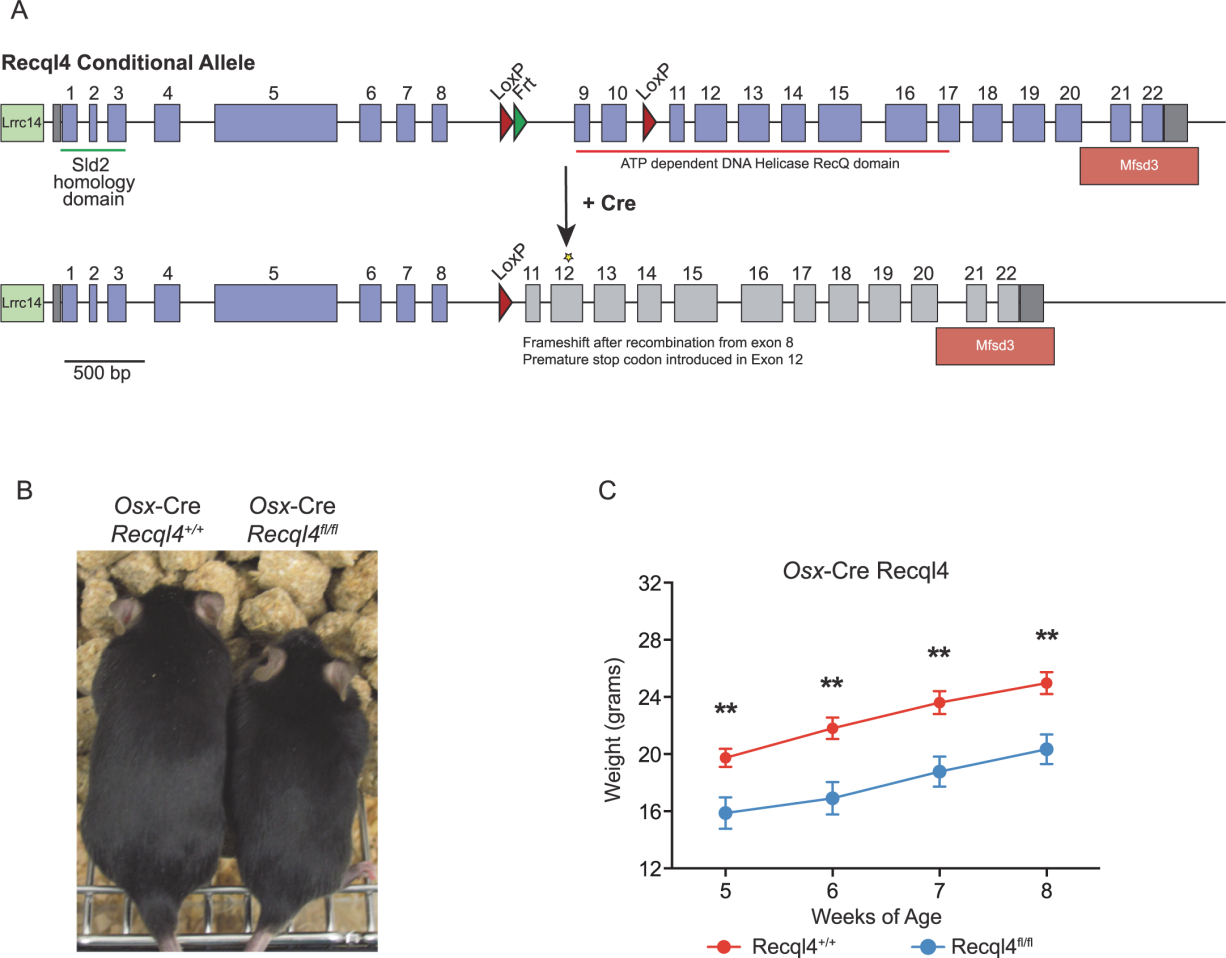
Deletion of Recql4 in osteoblast precursors results in dwarfism. (A) Diagram of Cre-mediated Recql4 deletion. (B) Representative photograph of male *Osx*-Cre *Recql4* ^*+/+*^ and *Recql4* ^*fl/fl*^ mice. (C) Gross body weights of male *Osx*-Cre *Recql4* mice. n ≥ 7 for both genotypes. Data presented as mean±SEM. **P<0.01 compared with *Recql4* ^*+/+*^, t-test.

As a result of the embryonic lethality of germ-line *Recql4* ^-/-^ animals and the lethal, non-skeletal phenotype upon somatic deletion of *Recql4* in adult mice [27], we moved to a lineage restricted deletion model to examine the role of Recql4 in skeletal development and malignancy. To achieve osteoblast-restricted deletion of *Recql4*, we crossed the *Recql4* ^*fl/fl*^ mice with two Cre strains active at distinct stages of osteoblast development (Fig 1A). Firstly, we used the *Osterix1-GFP*::*Cre* (*Osx*-Cre) transgenic mouse where Cre is active in the proliferative osteoblast progenitor populations [28]. Secondly, to delete *Recql4* in the committed mature osteoblast/osteocyte population we used the Dentin matrix protein 1-Cre (*Dmp1*-Cre) transgenic line [29]. In both cases, the *Rosa26*-eYFP strain that reports Cre activity through the expression of eYFP was included to allow for tracing and isolation of Cre exposed cells [30]. The distinct spatiotemporal expression of *Osx* and *Dmp1* allows for *in vivo* assessment of the requirement for *Recql4* in osteoblast lineage development.

### Reduced skeletal growth in *Osx*-Cre *Recql4* ^*fl/fl*^ mice

It had previously been reported that heterozygous germ-line *Recql4* mutant mice displayed a skeletal phenotype, although their human counterparts (parents/siblings of RTS patients) do not present with any apparent skeletal changes [3,27,31]. We assessed skeletal parameters by micro-computed tomography (μCT) in male germ-line exon 9–10 deficient *Recql4* ^*+/-*^ mutants. We observed no significant changes in any bone parameter, nor did we observe any phenotype (malignant or otherwise) in the *Recql4* ^*+/-*^ mice upon extended aging.

**Fig 2:**
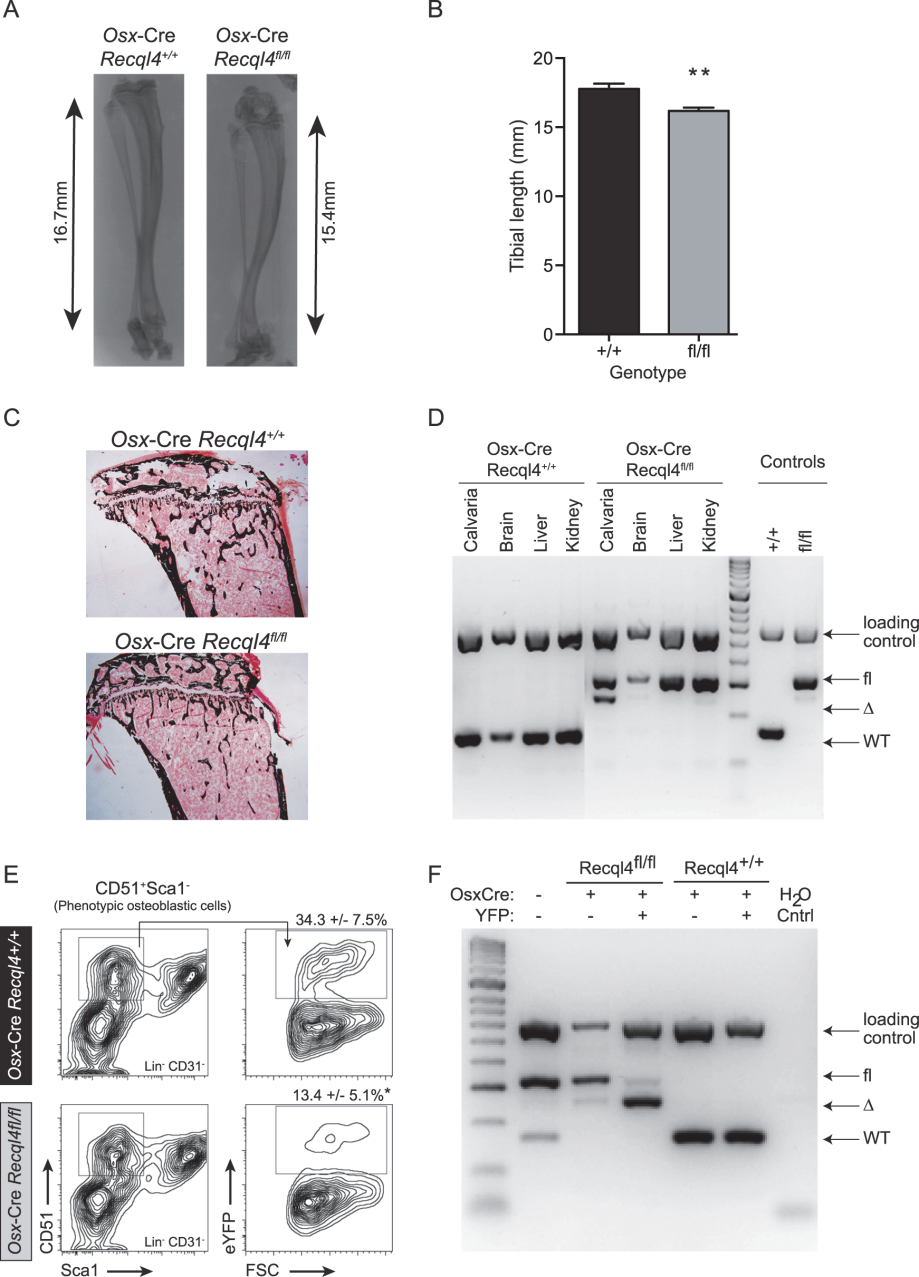
*Osx*-Cre *Recql4* ^*fl/fl*^ mice have reduced bone length. (A) X-ray of tibia from male *Osx*-Cre *Recql4* ^*+/+*^ and *Recql4* ^*fl/fl*^ mice. (B) Measurement of tibial length from *Osx*-Cre *Recql4* ^*+/+*^ and *Recql4* ^*fl/fl*^ mice. n≥ 9 for both genotypes. Data presented as mean±SEM. **P<0.01 compared with *Recql4* ^*+/+*^, t-test. (C) Representative photo of Von Kossa staining of 9 week old *Osx*-Cre *Recql4* tibiae. (D) Detection of Recql4 genomic excision in various tissues from *Osx*-Cre mice. (E) Percentage of FACS-sorted osteoblastic cells (Lin^-^ CD31^-^ CD51^+^ Sca1^-^) from *Osx*-Cre *Recql4* mice. Data presented as mean±SEM. *P<0.05 compared with *Recql4* ^*+/+*^, t-test. (F) Osteoblastic cells from *Osx*-Cre R26YFP *Recql4* mice were sorted into eYFP+ve and eYFP-ve fractions prior to genomic DNA extraction and assessment of Recql4 genomic excision.

We next investigated the effects of *Recql4* deletion from the pre-osteoblast populations using *Osx*-Cre on normal skeletal development. Only male mice were assessed for all genotypes to exclude any influence of sex differences in bone structure, and all controls in these studies are *Osx*-Cre+ *Recql4* ^*+/+*^ to control for the effects of the transgene alone on bone [32,33]. While *Osx*-Cre *Recql4* ^*fl/+*^ had no phenotype and were no different to *Osx*-Cre *Recql4* ^*+/+*^ controls, *Osx*-Cre *Recql4* ^*fl/fl*^ mice were smaller than *Osx*-Cre *Recql4* ^*+/+*^ control littermates as evidenced by a significantly lower body weight (Fig 1B and 1C). *Osx*-Cre *Recql4* ^*fl/fl*^ mice had shorter tibia at 9 weeks of age (Fig 2A and 2B), while there was less mineralized bone within the long bones when assessed by von Kossa staining (Fig 2C). Although efficient genomic recombination of *Recql4* in adult tissues was only observed in bone tissue (Fig 2D), we had previously observed a selection against stable deletion of *Recql4* in hematopoietic cells [27]. To ascertain if stable deletion was occurring in osteoblast precursors, we isolated hematopoietic and vascular marker negative (CD45^-^Lin^-^CD31^-^) cells from the collagenase digested compact long bones. These were fractionated into eYFP positive and negative populations, distinguishing cells that have expressed Cre during development and would be expected to have *Recql4* recombined (eYFP+ve), from Cre negative cells. Genomic DNA was isolated from each population from *Osx*-Cre *Recql4* ^*fl/fl*^ and *Osx*-Cre *Recql4* ^*+/+*^ control littermates. Essentially complete deletion of exons 9 and 10 was observed in the eYFP+ve fraction from the *Osx*-Cre *Recql4* ^*fl/fl*^ (Fig 2E and 2F). This demonstrates that stable deletion was achieved with no recovery of non-deleted/heterozygous cells to confound the interpretation of the phenotype. Nonetheless, during these cell isolations a significantly lower proportion of eYFP positive phenotypic osteoblastic cells was noted in the *Osx*-Cre *Recql4* ^*fl/fl*^ mice compared to controls (Fig 2E and 2F).

**Fig 3:**
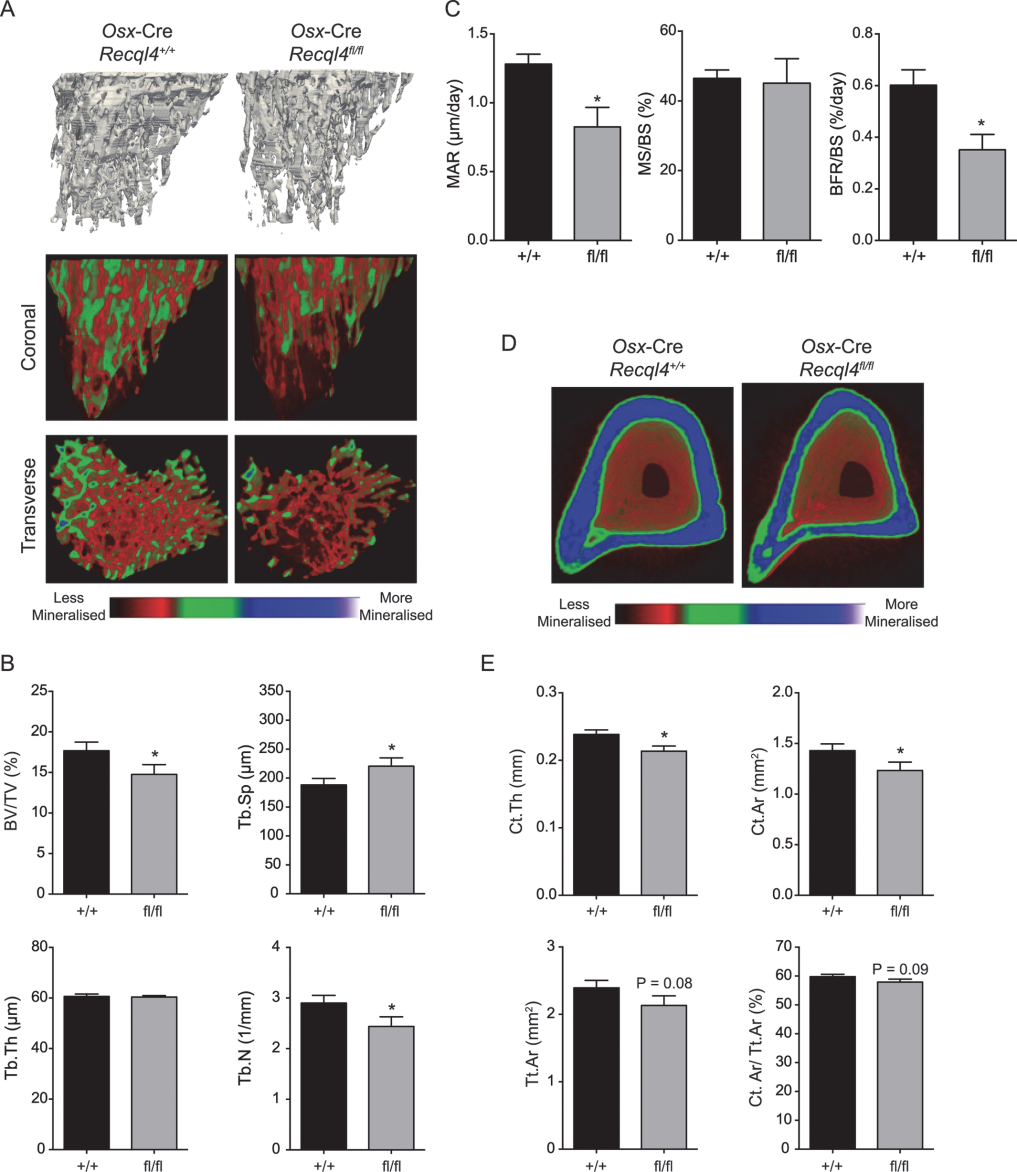
*Osx*-Cre *Recql4* ^*fl/fl*^ mice have a low bone mass. (A) Representative images of reconstructed trabecular region of the proximal tibial secondary spongiosa (top) and color-coded quantitative mineralization images (bottom) from male *Osx*-Cre *Recql4* ^*+/+*^ and *Recql4* ^*fl/fl*^ mice. (B) Trabecular bone volume (BV/TV), trabecular separation (Tb.Sp), trabecular number (Tb.N), trabecular thickness (Tb.Th) of *Osx*-Cre *Recql4* tibiae. n≥ 8 for both genotypes. (C) Mineralization apposition rate (MAR), mineralizing surface per unit bone surface (MS/BS) and bone formation rate per unit bone surface (BFR/BS) from the dynamic histomorphometric analysis of the proximal tibial secondary spongiosa. n = 5–6 for both genotypes. (D) Representative images of reconstructed cortical bone with colour-coded quantitative mineralisation from *Osx*-Cre *Recql4* ^*+/+*^ and *Recql4* ^*fl/fl*^ mice. (E) Cortical thickness (Ct.Th), cortical bone area (Ct. Ar), total cortical cross-sectional bone area (Tt.Ar) and cortical bone area fraction (Ct.Ar/Tt.Ar) of *Osx*-Cre *Recql4* cortical tissue. n≥ 8 for both genotypes. Data presented as mean±SEM. *P<0.05, compared with *Recql4* ^*+/+*^, t-test.

The effect of *Recql4* deletion from the early stages of osteoblast differentiation on skeletal architecture was assessed by μCT analysis of the trabecular bone within the secondary spongiosa and the diaphyseal cortical bone of the tibia. Qualitative assessment of pseudo-colored volume-rendered images of *Osx*-Cre *Recql4* ^*fl/fl*^ tibiae showed an apparently lower density with less trabecular bone (Fig 3A). This trabecular phenotype presented with a 17% reduction in bone volume as a proportion of total volume (BV/TV) (Fig 3B) compared to controls. Correspondingly, there was a 16% decrease in trabecular number (Tb.N) and a 17% increase in trabecular separation (Tb.Sp.), with no change in trabecular thickness (Tb.Th) (Fig 3B). We further characterized the bone structure and remodeling by histomorphometry within the proximal secondary spongiosa of the tibia. The low trabecular bone mass observed by μCT was associated with similar, but not statistically significant reductions in total bone volume and trabecular number, and increased trabecular separation in this second site (S1A Fig). There was no change in osteoid surface, thickness or volume, nor in the numbers of osteoblasts (N.Ob/B.Pm) or osteoclasts (N.Oc/B.Pm) per unit of bone perimeter (S1C Fig). Despite this, dynamic histomorphometry showed a significant 36% and 42% decrease in both mineral apposition rate (MAR) and bone formation rate (BFR/BS) respectively in *Osx*-Cre *Recql4* ^*fl/fl*^ mice, with no change in the mineralising surface per bone unit surface (MS/BS) compared to control mice (Fig 3C). Within the cortical bone, *Osx*-Cre *Recql4* ^*fl/fl*^ tibiae showed 10% and 14% reduction in cortical thickness (Ct.Th) and cortical bone area (Ct.Ar) respectively, with slight, but non-significant decrease in cortical cross-sectional bone area (Tt.Ar) and cortical bone area fraction (Ct.Ar/Tt.Ar) in the *Recql4* deficient animals (Fig 3D and 3E). Collectively, these data demonstrate an essential requirement for *Recql4* in osteoblast function.

Given the known role of the bone marrow microenvironment in regulating hematopoietic homeostasis, we also assessed hematopoiesis in the *Osx*-Cre *Recql4* ^*fl/fl*^ and *Osx*-Cre *Recql4* ^*+/+*^ littermates. We detected no differences in peripheral blood composition nor bone marrow hematopoiesis in the osteoblast restricted *Recql4*-deficient animals (S2A–S2C Fig). Recql4 deficient osteoblasts are able to support hematopoiesis normally.

### The deletion of *Recql4* in mature osteoblastic cells did not result in a bone phenotype

**Fig 4:**
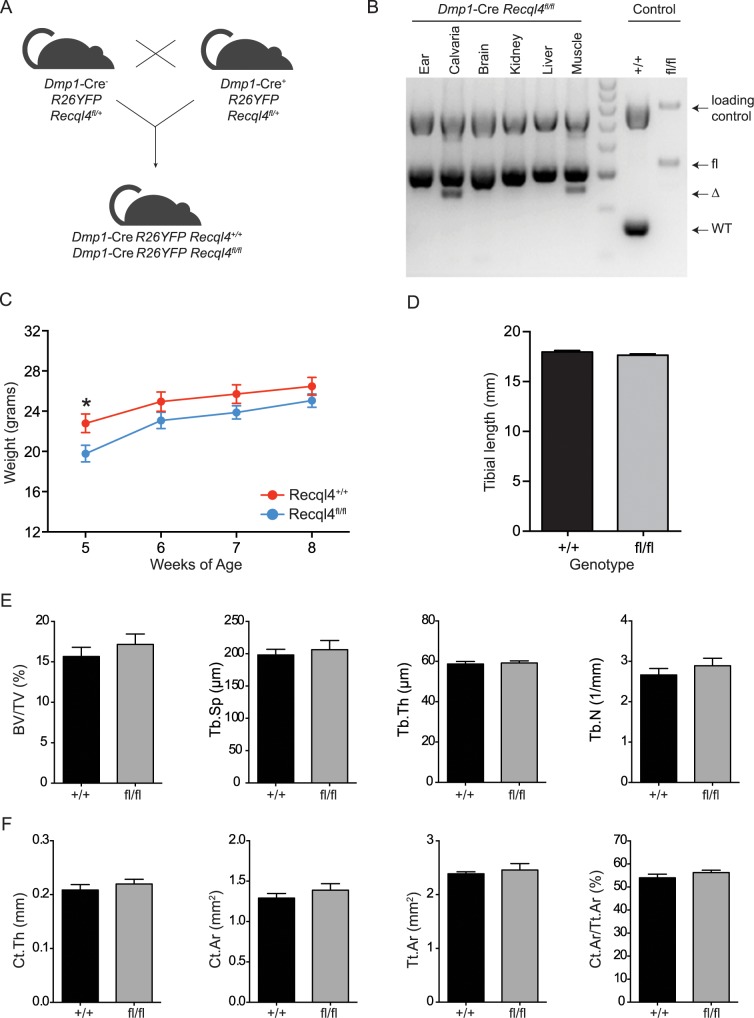
*Dmp1*-Cre *Recql4* ^*fl/fl*^ mice do not display a bone phenotype. (A) Schematic of generating *Dmp1*-Cre *Recql4* ^*+/+*^ and *Recql4* ^*fl/fl*^ mice. (B) The assessment of Recql4 genomic excision in various tissues from *Dmp1*-Cre mice. (C) Gross body weights of male *Dmp1*-Cre Recql4 mice. n = 4 for *Recql4* ^*+/+*^, n = 6 for *Recql4* ^*fl/fl*^. (D) Measurement of tibiae from *Dmp1*-Cre *Recql4* ^*+/+*^ and *Recql4* ^*fl/fl*^ mice. n = 4 for *Recql4* ^*+/+*^, n = 8 for *Recql4* ^*fl/fl*^. (E) Trabecular bone volume (BV/TV), trabecular separation (Tb.Sp), trabecular number (Tb.N), trabecular thickness (Tb.Th) of *Dmp1*-Cre *Recql4* tibiae. (F) Cortical thickness (Ct.Th), cortical bone area (Ct. Ar), total cortical cross-sectional bone area (Tt.Ar) and cortical bone area fraction (Ct.Ar/Tt.Ar) of *Dmp1*-Cre *Recql4* cortical tissue. Data from 9 week old male mice; n = 4 for *Recql4* ^*+/+*^, n = 8 for *Recql4* ^*fl/fl*^. Data presented as mean±SEM. *P<0.05 compared with *Recql4* ^*+/+*^, t-test.

Given the striking effect of *Recql4* deletion from pre-osteoblast stages on skeletal development, we sought to determine what impact deletion of *Recql4* would have when restricted to mature osteoblasts and osteocytes. To this end, we used the *Dmp1*-Cre transgenic line [29,34] (Fig 4A). Efficient genomic deletion of *Recql4* was seen in bone tissues, however unlike *Osx*-Cre, we also observed genomic recombination of *Recql4* in muscle tissue, as previously reported for this Cre line (Fig 4B) [35]. Littermate male *Dmp1*-Cre *Recql4* ^*+/+*^ and *Dmp1*-Cre *Recql4* ^*fl/fl*^ mice were comparable in weight and overall size (Fig 4C) and tibial lengths were the same between the cohorts (Fig 4D). Analysis by μCT of the trabecular and cortical bone regions of the tibia did not demonstrate any differences in those parameters assessed (Fig 4E and 4F). Collectively, the data indicate that although *Recql4* is required in the proliferating and differentiating osteoblastic population, it is not required in mature osteoblast and osteocyte populations to maintain bone homeostasis.

### Depletion of *Recql4* resulted in inhibition of cell proliferation and osteogenic differentiation

We initially planned to use FACS-purified, eYFP positive osteoblastic cells from the *Osx*-Cre *Recql4* ^*+/+*^ and *Osx*-Cre *Recql4* ^*fl/fl*^ mice to delineate the role of *Recql4* (Fig 2E). However, the low yields and very poor proliferation of the eYFP positive cells from *Osx*-Cre *Recql4* ^*fl/fl*^ cells rendered them unsuitable for further analysis [36,37]. We therefore utilized primary long bone osteoblastic cells from *Rosa26*-CreER^T2^ *Recql4* mice [27]. Tamoxifen (4-OHT) was added to these cultures for up to 21 days to activate Cre mediated deletion of *Recql4* and cell proliferation assays were performed during the first and third week.

**Fig 5:**
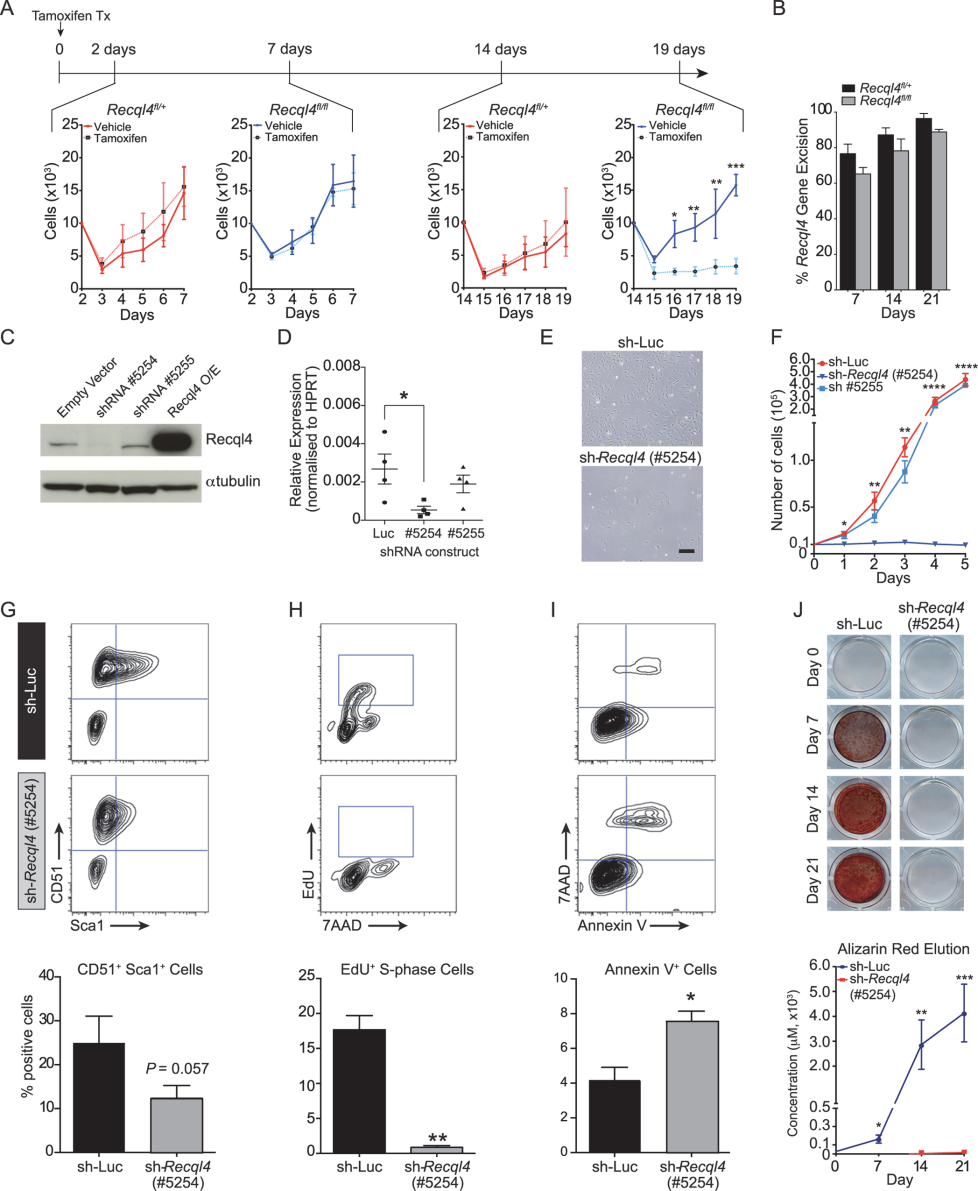
The depletion of *Recql4 in vitro* causes proliferation arrest. (A) Primary long bone osteoblastic cells were derived from *Rosa26*-CreER^T2^ *Recql4* mice treated with tamoxifen and subjected to proliferation assays. n = 3 independent cultures per genotype. (B) The assessment of Recql4 genomic excision from tamoxifen-treated osteoblastic cells. n = 3 independent cultures per genotype. (C) Immuno-blot of Recql4 protein levels from Kusa4b10 cells, with α-tubulin as a loading control. (D) Quantitative real-time PCR (qPCR) analysis of *Recql4* from Kusa4b10 cells. Relative gene expression levels were normalised to the expression of *Hprt*. n = 4 biological replicates. (E) Light microscopic images of control (shRNA-Luc) and sh-*Recql4* (#5254) Kusa4b10 cells (200X magnification). (F) shRNA-Luc and sh-Recql4 (#5254) cells were seeded in 6-well plates and cell counts were performed at 24hr intervals. n = 4 independently infected cultures. (G-I) Control (shRNA-Luc) and sh-*Recql4* (#5254) cells were seeded 24 hrs prior to flow cytometry analysis, and were stained with antibodies against CD51 and Sca1 (G), EdU and 7AAD (H), and Annexin V and 7AAD (I). Representative FACS plots shown on the top, percentages of stained cells summarised in bar graphs at bottom. n = 3–4 biological replicates. (J) shRNA-Luc and sh-*Recql4* (#5254) cells were subjected to osteogenic differentiation and stained with Alizarin Red. Representative photos are shown at top, while the quantification of eluted Alizarin Red dye is shown at the bottom. n = 3 biological replicates; All graphical data presented as mean±SEM. *P<0.05, **P<0.01, ***P>0.0005, ****P>0.00001 compared with control, t-test or 2-way ANOVA.

*Rosa26*-CreER^T2^ *Recql4* ^*fl/+*^ cells (control, referred to as *Recql4Δ/+)* proliferated comparably to non-tamoxifen treated cultures for the entire culture period (21 days). When assessed 7 days after the addition of tamoxifen, gene recombination was apparent in the *Rosa26*-CreER^T2^ *Recql4* ^*fl/fl*^ cells (homozygous deletion of *Recql4*, referred to as *Recql4Δ/Δ)*, but only slightly more than 60% of the floxed allele had been deleted. Between days 2 and 7, there was no difference in proliferation rates between *Recql4Δ/+* and *Recql4Δ/Δ* cells (Figs 5A, 5B and S3). At day 14 after tamoxifen addition, recombination of the Recql4 allele had increased to closer to 80% in both sets of genotypes (Figs 5B and S3). At this time, the tamoxifen-treated *Recql4 Δ/Δ* primary osteoblasts failed to proliferate (Fig 5A), while non-tamoxifen treated *Recql4Δ/Δ* cells proliferated similarly to controls (*Recql4Δ/+* ± tamoxifen). The more complete the genomic deletion of *Recql4*, the more profound the proliferation arrest was apparent (Figs 5B and S3). These studies demonstrate a requirement for *Recql4* in normal osteoblast expansion, and suggest that the *in vivo* phenotype may be attributed to the impaired proliferation of *Recql4* deficient osteoblastic cells.

To allow for a more detailed analysis of osteoblast proliferation and differentiation, we moved to a short hairpin RNA (shRNA) approach. We utilized the Kusa4b10 murine cell line which has the capacity to differentiate into osteoblastic or adipogenic lineages [38]. We identified one shRNA (#5254), amongst numerous screened, which reduced the expression of *Recql4* at both the transcript and protein levels in Kusa4b10 cells (Figs 5C, 5D, S4A, and S4B). Controls were either a second shRNA against Recql4 (#5255) that did not reduce transcript levels, or a luciferase-targeting shRNA.

The knockdown of *Recql4* caused a striking phenotype in the Kusa4b10 cells. In particular, the cells displayed a ‘flattened’ morphology as compared to the controls, and proliferation was completely blocked (Fig 5E and 5F). The cell surface phenotype of the knockdown cells changed, with a reduction in the expression of Sca-1, a marker of proliferation, but no change in CD51 expression (Fig 5G) [39,40]. Depletion of *Recql4* led to a 2-fold increase in apoptosis, as evidenced by increased Annexin V staining (Fig 5I). The most pronounced difference, however, became apparent when we assessed the cell cycle distribution of *Recql4* knockdown cells. EdU pulse-labelling demonstrated a near complete absence of DNA replication or S-phase populations in the knock-down cells compared to controls (Fig 5H). In contrast to recent reports that assessed the acute depletion of RECQL4 with shRNA or siRNA [41], we did not observe an increase in senescence associated ß-galactosidase positive cells in *Recql4*-knockdown cultures compared to control infected cultures when assessed at similar time points post knockdown (S4C and S4D Fig). Taken together with the primary osteoblast analysis, these *Recql4* depletion leads to a profound proliferation arrest and an elevated level of apoptosis without appreciable effects on senescence.

To determine if the loss of *Recql4* affected osteoblast differentiation in addition to proliferation, control and knock-down Kusa4b10 cells were placed under osteoblastic differentiation conditions. To account for the reduced proliferative potential of the *Recql4* depleted cells, the cells were seeded to be fully confluent at the start of differentiation. The knockdown of *Recql4* led to a near complete failure in mineralization, as assessed by alizarin red staining, over a 21 day differentiation time-course (Fig 5J). All wells were visually confirmed to have a confluent sheet of cells, yet the *Recql4* depleted cells were unable to mineralise under these assay conditions. Profiling of markers of osteoblastic maturation and differentiation by qPCR revealed that whilst *Runx2* expression was not affected, all markers of osteoblast differentiation downstream of this were significantly reduced in *Recql4* knockdown cells, consistent with the failure to mineralize (S4E Fig). These data suggest that *Recql4* is not only required for normal proliferation of osteoblast precursors but also contributes to their maturation *in vitro*, a result consistent with the low bone formation rate in the presence of unchanged osteoblast numbers in the *Osx*-Cre *Recql4* ^*fl/fl*^ mice.

### The concurrent loss of *p53* does not rescue *Recql4* deficient osteoblast proliferation

**Fig 6:**
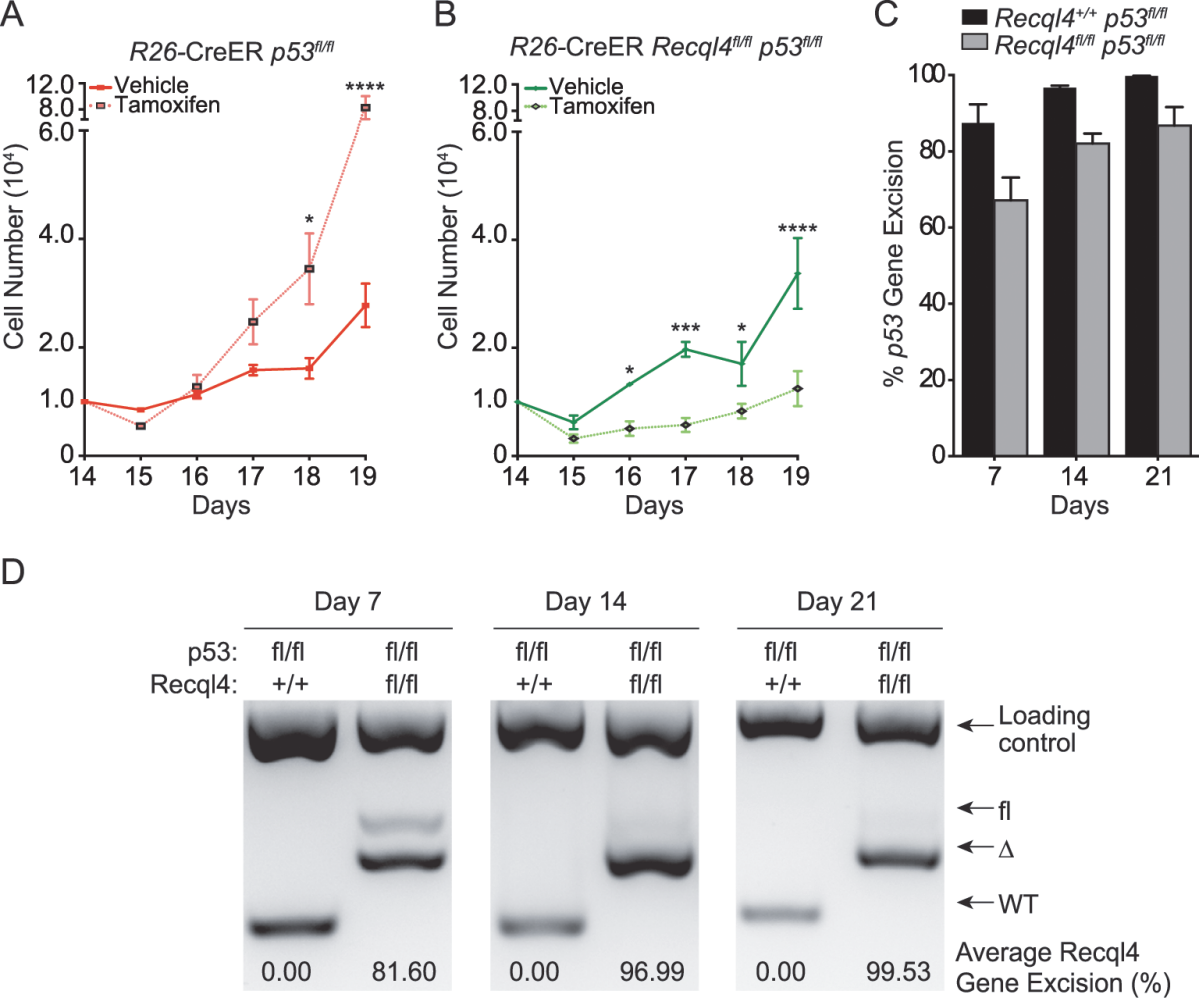
Concurrent loss of p53 does not modify the Recql4 phenotype. (A) and (B): Primary long bone osteoblastic cells were derived from *R26*-CreER^T2^ *Recql4 p53* mice. They were then treated with tamoxifen and subjected to proliferation assays. n = 3–4 independent cultures per genotype; Data presented as mean±SEM. *P<0.05, **P<0.01, ***P>0.0005, ****P>0.00001 compared with vehicle, 2-way ANOVA. (C) The assessment of p53 genomic excision from tamoxifen-treated long bone osteoblastic cells from *R26*-CreER^T2^ *Recql4 p53* mice. Data presented as mean±SEM. (D) The assessment of Recql4 genomic excision from tamoxifen-treated long bone osteoblastic cells from *R26*-CreER^T2^ *Recql4 p53* mice.

Recql4 has been proposed to interact with p53, which has been reported to inhibit osteoblast proliferation [15,42]. To explore the relationship between the two genes, we treated primary long bone osteoblastic cells from *R26*-CreER^T2^ *Recql4* ^*fl/fl*^ *p53* ^*fl/fl*^ mice with tamoxifen and subjected them to proliferation assays as described previously (Fig 6A). After 14 days, the *Recql4* ^+/+^ *p53Δ*/*Δ* cells displayed increased proliferation compared to its non-tamoxifen treated isogenic counterpart. In contrast, concurrent and equally efficient, deletion of both *Recql4* and *p53*, resulted in proliferation kinetics similar to the *Recql4Δ/Δ* single mutants (Figs 4A and 6B–6D). This demonstrated that the loss of p53 is insufficient to rescue the proliferative defect of *Recql4Δ/Δ* osteoblasts *in vitro*.

### *Osx*-Cre *Recql4* ^*fl/fl*^ mice do not spontaneously develop osteosarcoma

**Fig 7:**
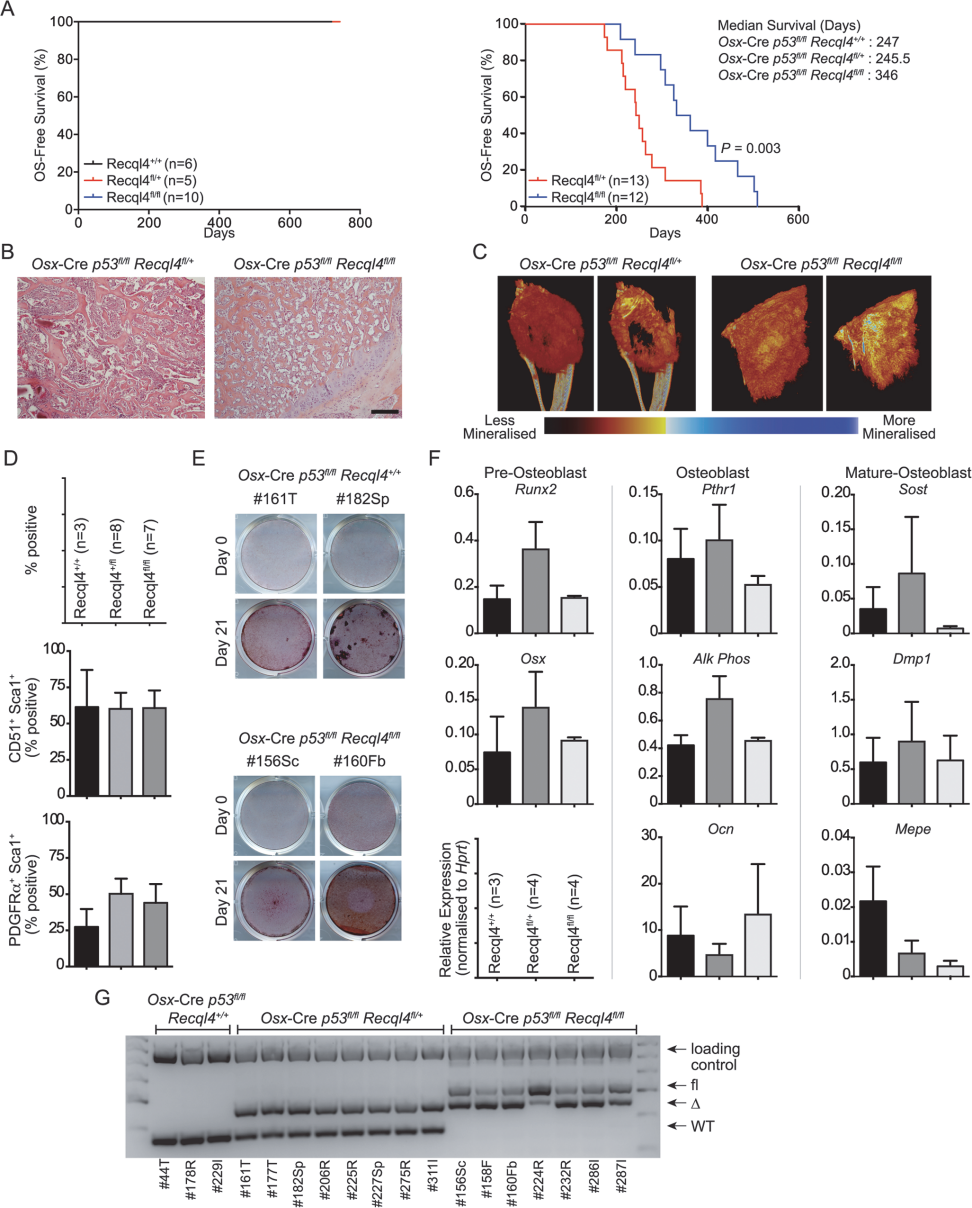
The loss of Recql4 does not initiate osteosarcoma. (A) Kaplan-Meier survival plots of *Osx*-Cre *Recql4* (left) and *Osx*-Cre *p53* ^*fl/fl*^ *Recql4* (right) mice. P value calculated by Log-Rank statistical test. (B) H&E stained sections of primary OS tumors from *Osx*-Cre *p53* ^*fl/fl*^ *Recql4* animals of indicated genotype. (C) Representative reconstructed μCT images of primary OS tumors from *Osx*-Cre *p53* ^*fl/fl*^ *Recql4* ^*fl/+*^ and *Recql4* ^*fl/fl*^ mice. (D) Flow cytometry percentages of tumor cells stained with CD51, Sca1 and PDGFRα. n = 3 for *Recql4* ^*+/+*^, n = 8 for *Recql4* ^*fl/+*^, n = 7 for *Recql4* ^*fl/fl*^; Data presented as mean±SEM. (E) Representative photos of Alizarin Red-stained tumor cells that were subjected to osteogenic differentiation conditions. (F) qPCR profiling of *Osx*-Cre *p53* ^*fl/fl*^ *Recql4* ^*+/+*^, *Recql4* ^*fl/+*^ and *Recql4* ^*fl/fl*^ tumors for the indicated genes. n = 3–4; Data presented as mean±SEM. (G) Assessment of genomic excision of Recql4 in tumor-derived cell lines. 40ng of genomic DNA was used for PCR and subjected to gel electrophoresis.

Given the high incidence of OS in RTS patients, we established cohorts of *Osx*-Cre *Recql4* ^*+/+*^, *Osx*-Cre *Recql4* ^*fl/+*^ and *Osx*-Cre *Recql4* ^*fl/fl*^ mice that were allowed to age and were monitored for the development of cancer, particularly OS. These cohorts were followed over a 100 week period. No animals were found with OS, irrespective of the genotype (Fig 7A and S1 Table). Whilst we may not have a sufficiently large sample size to resolve a low incidence of OS development within these specific cohorts, no OS has ever been observed in the *Osx*-Cre *Recql4* ^*fl/fl*^ mice within our entire colony (>100 *Osx*-Cre *Recql4* ^*fl/fl*^ mice of various ages up to 2 years). This demonstrates that complete deletion of *Recql4* in osteoblast precursors is not sufficient to initiate OS in the mouse at high frequency.

### Increased OS-free survival in *Osx*-Cre *p53* ^*fl/fl*^ *Recql4* ^*fl/fl*^ double knock-outs

The only mutation to occur in all human OS is the loss or mutation of *TP53* [12]. Experimentally, loss of *p53* from within the osteoblast lineage is sufficient to initiate OS with near complete penetrance [14,17]. To determine whether deletion of *Recql4* in an *Osx*-Cre *p53* ^*fl/fl*^ background would accelerate OS initiation and shorten OS-free survival, we established cohorts of *Osx*-Cre *p53* ^*fl/fl*^ *Recql4* ^*+/+*^, *Osx*-Cre *p53* ^*fl/fl*^ *Recql4* ^*fl/+*^ and *Osx*-Cre *p53* ^*fl/fl*^ *Recql4* ^*fl/fl*^ mice. OS was observed the in *Osx*-Cre *p53* ^*fl/fl*^ *Recql4* ^*+/+*^ mice as expected with a median onset of 247 days (Fig 7A and S1 Table; larger comparison cohort from our colonies previously reported: median survival time 226 days [43]). The loss of one allele of *Recql4* did not significantly affect OS-free survival time compared to the *Osx*-Cre *p53* ^*fl/fl*^ *Recql4* ^*+/+*^ mice, consistent with the lack of phenotype in *Recql4* heterozygous animals and humans (Fig 7A and S1 Table). However, and unexpectedly, *Osx*-Cre *p53* ^*fl/fl*^ *Recql4* ^*fl/fl*^ mice had a significantly increased OS-free survival, with a median survival time of 346 days compared to *Osx*-Cre *p53* ^*fl/fl*^ *Recql4* ^*fl/+*^ controls (Fig 7A and S1 Table).

The *Osx*-Cre *p53* ^*fl/fl*^ *Recql4* ^*fl/fl*^ mice more frequently presented with multiple primary tumors as compared to the *Recql4* wildtype or heterozygous cohorts. The majority of the primary OS were distributed in axial locations (eg., the rib-cage and the vertebrae). We have not observed any non-OS tumors, such as hibernoma, in these cohorts independent of the genotype. Interestingly, while there was no difference in macroscopic metastatic incidence between *Recql4* ^*+/+*^ and *Recql4* ^*fl/fl*^ tumour-bearing mice (including comparison to larger historic cohorts within the same facility), the *Recql4* ^*fl/+*^ mice presented with an increased metastatic incidence at autopsy compared to either the *Osx*-Cre *p53* ^*fl/fl*^ *Recql4* ^*+/+*^ or *Osx*-Cre *p53* ^*fl/fl*^ *Recql4* ^*fl/fl*^ mice. Irrespective of *Recql4* status, metastatic tumors were most commonly located in the lung.

To understand if loss of *Recql4* altered the biology of the OS that arose, we analysed a range of tumor characteristics. There were no grossly apparent differences between the three cohorts in the histology of the OS as assessed by either histology or μCT scans of primary tumors (Figs 7B, 7C and S5). Cell surface markers PDGFRa, CD51 and Sca-1 were screened on primary cell cultures derived from tumors of the individual genotypes and there was no difference based on *Recql4* status (Fig 7D). The OS cell cultures behaved similarly when placed under differentiation inducing culture conditions (Fig 7E). There were no differences in the expression of a panel of osteoblast differentiation stage markers as assessed by qPCR (Fig 7F). Therefore, the only appreciable differences between OS arising in the *Osx*-Cre *p53* ^*fl/fl*^ *Recql4* ^*fl/+*^ and *Osx*-Cre *p53* ^*fl/fl*^ *Recql4* ^*fl/fl*^ mice was the survival time and metastatic frequency. The explanation for the increased OS-free survival in the double knock-outs became apparent when we assessed the status of the *Recql4* allele in the OS cells. In all cases, complete genomic recombination of the single floxed *Recql4* allele was seen in the OS from *Osx*-Cre *p53* ^*fl/fl*^ *Recql4* ^*fl/+*^ mice. In striking contrast, in no case did we observe complete deletion of both floxed *Recql4* alleles in the *Osx*-Cre *p53* ^*fl/fl*^ *Recql4* ^*fl/fl*^ mice (Fig 7G). This demonstrates that mutations resulting in null alleles of *Recql4* are not initiating events in OS and that loss of *Recql4* does not co-operate with *p53* mutation to initiate OS. The increased OS-free survival *Osx*-Cre *p53* ^*fl/fl*^ *Recql4* ^*fl/fl*^ mice is most likely a result of selective pressure to retain an intact allele of *Recql4* in the tumor that arises.

## Discussion

In humans, mutations of RECQL4 are associated with a series of related syndromes RTS, RAPADILINO Syndrome and Baller-Gerold Syndrome. In common amongst these syndromes, and other human disorders associated with mutations in RecQ helicases, are defects in skeletal homeostasis leading to skeletal abnormalities including low bone mass, and in the case of RTS, OS [1]. Here we demonstrate that *Recql4* is required for the expansion and normal differentiation of osteoblast precursor populations. *In vivo* we demonstrated that the deficiency of *Recql4* in *Osx*-Cre expressing osteoblastic cells resulted in shorter bones and reduced bone mass (trabecular and cortical) that resulted from a reduced bone formation rate. The absence of changes in osteoclastogenesis as assessed by histomorphometry, and hematopoiesis more generally, indicate that the phenotype was osteoblast cell intrinsic. When deletion of *Recql4* was restricted to more mature populations using *Dmp1*-Cre, the mice did not demonstrate a skeletal phenotype. Therefore, *Recql4* is required for the normal proliferation and expansion of the pre-osteoblast populations, but it is dispensable for mature osteoblasts/osteocytes, including the functions of osteocytes that regulate mineralization. At the resolution of histomorphometry it is only possible to describe the nature of the cells (osteoblast lineage etc) not whether they are still proliferative or viable, as we have described in other mutants [36]. As *Osx*-Cre expressing cells turn over regularly [44] it is as expected that a static view of osteoblasts per bone surface may not yield a difference but the overall bone formation rate and amount of bone in the entire limb are reduced. This is consistent with a quantitative rather than a qualitative defect. In primary osteoblast cultures with induced deletion of Recql4 we saw normal proliferation for the first 14 days of culture and then suppressed proliferation in the *Recql4Δ/Δ*. This is very similar to the phenotype we previously reported in primary B- and T-cell cultures [27]. In keeping with a dilutional model of Recql4 function in DNA replication, as demonstrated in *Xenopus* [45], cells null for the *Recql4* gene proliferate normally for 2 weeks *in vitro* and for multiple weeks *in vivo* continue to contribute to bone formation. However, once they reach critically low levels of Recql4 protein they most likely cease proliferation and, ultimately, undergo apoptosis. Concurrent deletion of *p53* was not able to rescue the proliferative defect observed in *Recql4*-deficient cells. This is consistent with the failure of *p53* deletion to rescue the lethal bone marrow failure that occurs upon widespread somatic deletion of *Recql4* in adult mice [27]. Collectively, these studies lead us to conclude that the primary physiological function of *Recql4* is in cell proliferation and DNA replication [46,47].

*RECQL4* has well characterized roles in several critical cellular functions including DNA replication and genome stability [1,2,47,48]. Additional roles have been proposed based on studies in cell culture models including regulation of senescence, interactions with the mitochondria and p53 [42,49]. Our studies of osteoblast development and prior work on hematopoiesis are relevant to our understanding of the normal *in vivo* function of *Recql4*. The most prominent feature of *Recql4* deficiency, as opposed to hypomorphic mutation, is a failure in cell proliferation. In the hematopoietic system, a rapid fully penetrant bone marrow failure results from the deletion of *Recql4* [27]. The concurrent deletion of *p53* did not alter the hematopoietic failure, consistent with a primary DNA replication/S-phase cell cycle defect. We show that *in vivo* deletion of *Recql4* from proliferative pre-osteoblast populations, but not mature osteoblasts/osteocytes, caused a low bone mass phenotype. In primary osteoblast cultures, loss of Recql4 led to a failure to proliferate once gene deletion had efficiently occurred. As with hematopoiesis, *p53* deletion did not rescue the proliferative failure associated with *Recql4* deficiency in osteoblast cultures. We conclude that there is little evidence to support a direct genetic interaction between *Recql4* and *p53* pathways. This is supported by the recent data from Lu and colleagues who reported mild or no genetic rescue of a Recql4 deficient phenotype in the limb buds or growth plates by concurrent deletion of p53 [50]. The activation of *p53* in *Recql4* deficient cells most likely represents a secondary effect of impaired cell cycle progression and transcription, ultimately leading to apoptosis (S5C Fig). We did not find any increase in senescent cells as was recently reported in human fibroblasts infected with shRNA against *RECQL4*. It is possible that the mechanism that inhibits cell proliferation is different between the two cell types [41], but this seems less likely given the similarities observed in primary cell cultures of hematopoietic cells and osteoblasts.

The low trabecular and cortical bone volume of *Osx*-Cre *Recql4* ^*fl/fl*^ mice mirrors the low bone mass seen clinically in RTS patients [3–6]. However, unlike RTS and related patients, we did not see evidence of additional skeletal abnormalities such as polydactyly, radial ray defects or cleft palate as has been reported in *Recql4* hypomorphic mice [26]. The low bone mass phenotype is conserved and the severity of the skeletal abnormalities is likely impacted by two important differences between patient and mouse model data. Firstly, in patients, the mutant *RECQL4* proteins are present in all cells throughout the body and during all stages of skeletal development. Secondly, in the murine model, *Osx*-Cre does not delete *Recql4* from all bone forming cells. This results in a chimeric setting where *Recql4* wild-type cells are present and able to partially compensate for the deficiencies in bone formation that the *Recql4* null cells have. There are less *Recql4*-deficient cells in the bone as evidenced by the lower proportions of eYFP positive osteoblastic cells (Fig 2E). A completely *Recql4* deficient skeletal compartment would be expected to have a more profound phenotype. Consistent with this interpretation, it was recently reported that a more severe skeletal disturbance was achieved using *Prx1*-Cre to delete *Recql4* exons 5–8 [50,51]. This study was restricted to early developmental time points due to the impaired survival of the *Prx1*-Cre *Recql4* ^*fl/fl*^ animals and so no analysis of adult animals was reported. *Prx1-*Cre deletes in the early developing limb bud mesenchymal cells and leads to near complete deletion in the appendicular skeleton. The *Prx1*-Cre *Recql4* ^*fl/fl*^ mice had growth retardation and more severe limb developmental defects than those we observed with *Osx*-Cre. It should be noted that the *Osx*-Cre model we have used was not restricted in activity to the postnatal skeleton and is expressed throughout skeletal development. We therefore can not exclude a contribution from developmental effects or deletion in other mesenchymal populations, such as chondrocytes, to the adult bone phenotype that we describe. Therefore, our data and that of Lu and colleagues [50] indicate that the primary function of *RECQL4* in osteoblast precursors is to enable the appropriate expansion of cell populations required for normal skeletal formation. The low bone mass phenotype is consistent, albeit varying in severity, amongst different mesenchymal targeted Cre strains and Recql4 alleles. This analysis provides an explaination for the low bone mass that is a hallmark of *RECQL4* mutations in humans and our modeling demonstrates that this intrinsic function of *RECQL4* within the skeletal system.

RTS is notable as the third familial syndrome impacted by OS, in addition to Li-Fraumeni and hereditary retinoblastoma kindreds. However, unlike *TP53* and *RB1*, mutations in *RECQL4* are not a common feature of sporadic OS and appear restricted to RTS-related OS. A role of *Recql4* mutations in OS has not been revealed by previous mouse models because the viable mutants have hypomorphic *Recql4* alleles, leading to transcription of the the N-terminal regions [25,26]. One question that arises from these observations is whether OS in RTS patients represents the same disease as Li-Fraumeni and hereditary retinoblastoma kindred associated OS, and more importantly, sporadic OS in human. Our analyses demonstrate that the osteoblastic-restricted complete loss of function *Recql4* alleles are not able to initiate OS in the mouse. Indeed, we have not seen tumor formation in *Recql4* deficient animals or aged *Recql4* ^*+/-*^ animals of any type. The absence of OS formation in skeletally restricted Recql4 null mutants was also recently reported when Recql4 was deleted with *Prx1*-Cre [51]. *Prx1*-Cre deletes in the early limb bud mesenchymal cells and is active earlier than the *Osx*-Cre that we utilized. The results from the independent studies of an absence of OS initiation in Recql4 deficient skeletal models suggests that the failure to initiate OS does not reflect the choice of models, but rather, reflects the biology of Recql4 null alleles.

As RTS patients have a 30% incidence of OS this was somewhat unexpected, but not without precedence given our previous analysis of *Osx*-Cre *pRb* ^*fl/fl*^ animals which also do not develop OS despite the high rate of OS in hereditary retinoblastoma kindreds and the rate of *RB1* mutation in conventional OS [12,17]. Unexpectedly, homozygous deletion of *Recql4* delayed OS development in a fully penetrant *Osx*-Cre *p53* ^*fl/fl*^ model, directly contrasting with the acceleration of OS development when loss of *Rb* was combined with *p53* deletion. This observation was explained by the incomplete deletion of *Recql4* in the OS that did arise in the *Osx*-Cre *Recql4* ^*fl/fl*^ *p53* ^*fl/fl*^ mice. The increased OS-free survival is most likely because the *Osx*-Cre *p53* ^*fl/fl*^ *Recql4* ^*fl/fl*^ are unable to proliferate, a conclusion supported by our *in vitro* data, and the cell that ultimately gives rise to the tumor is being selected for incomplete deletion of the Recql4 locus. This is an inefficient process. This result demonstrates that OS most likely does not arise from the *Recql4* null cells, most plausibly due to the profoundly impaired proliferation of these cells. It further suggests that the OS initiating cells require some level, albeit low based on the analysis of RTS patients, of *RECQL4* function to enable OS initiation and maintenance [7]. The OS predisposition in RTS patients is most likely accounted for by the mutation spectrum in these patients resulting in the generation of C-terminal truncated RECQL4 proteins. These alleles are predicted to retain the essential role of *RECQL4* in DNA replication, but disable RecQ helicase dependent functions. Thus, our working hypothesis is that aneuploidy and tumor predisposition can only arise in cells retaining the Sld2-homology regions of RECQL4 where basal cell proliferation is intact but genomic stability is compromised [46,47,52]. Therefore, together with the present data, we conclude that the *RECQL4* is not an OS tumor suppressor when the mutation results in a null allele.

Our results demonstrate an essential, non-redundant role for *Recql4* in the expansion and proliferation of osteoblast precursors that is not required once the cells become mature osteoblasts. Complete null alleles of *Recql4* do not initiate OS and loss of *Recql4* does not potentiate OS initiated by *p53* deletion. That OS does not arise in *Recql4* null cells reflects an essential function for *Recql4* in the proliferation and expansion of the cells. These studies demonstrate that the low bone mass is a result of the loss of *RECQL4* function, but tumorigenesis most likely requires mutant *RECQL4* activity. The precise function of mutant RECQL4 in OS initiation remains to be defined. Collectively our study reveals that *Recql4* is essential for normal skeletal homeostasis and that the greatly elevated rates of OS in RTS patients are not recapitulated by the complete absence of *Recql4*.

## Materials and Methods

### Ethics statement

All animal experiments were approved by the Animal Ethics Committee of St Vincent’s Hospital, Melbourne. AEC #044/10.

### Mice

*Recql4* ^*fl/fl*^ mice (C57BL/6-*Recql4* ^*tm2272Arte*^) were generated by TaconicArtemis GmbH (Cologne, Germany); full details of the allele have been previously described [27]. *Osx*-Cre, *DMP1*-Cre, *Rosa26*-eYFP and *p53* ^*fl/fl*^ animals have been previously described and were on a C57Bl/6 background [17,28,29,53]. 5-week old male mice were weighed weekly over a 4-week period. All histomorphometry and microCT was done on 9 week old male mice.

### Histology and histomorphometry

Tumour tissue was obtained from *Osx*-Cre *Recql4 p53* ^*fl/fl*^ mice, and it was fixed in 2–4% paraformaldehyde overnight at 4°C. After embedding in paraffin, the tissue was sectioned and stained with hematoxlyin and eosin. Pathological assessment of the tumor sections was performed blinded to the sample genotype. For dynamic histomorphometry, *in vivo* labeling of bone formation was performed with intra-peritoneal injection of 8-week old male mice with alizarin (Sigma Aldrich) and calcein (Sigma Aldrich) 7 days and 2 days respectively before tissue collection [54]. Tibia were fixed in 4% paraformaldehyde and embedded in methylmethacrylate, 5μm sections were stained with von Kossa to identify mineralized tissue and toluidine blue for static histomorphometry [53,55]. Histomorphometric analysis was carried out in the secondary spongiosa of the proximal tibia metaphysis (Osteomeasure, Osteometrics, Atlanta, GA, USA) as previously described [56].

### MicroCT analysis of bone parameters

*Ex vivo* microCT was performed on tibiae and tumor material using the SkyScan 1076 system (Bruker-microCT, Kontich, Belgium). Images were acquired using the following settings: 9μm pixel size, 0.5mm aluminum filter, 50kV voltage and 100μA current, 2400 ms exposure time, rotation 0.5°, frame averaging = 1. Images were reconstructed and analysed with SkyScan software programs NRecon (version 1.6.3.3), DataViewer (version 1.4.4), CT Analyser (CTan, version 1.12.0.0) and CTVox (version 2.2.0). The trabecular analysis region of interest (ROI) was determined by identifying the start of the mineralized zone of the proximal growth plate and calculating 16.5% of the total tibial length towards the tibial mid-shaft, where we then analysed an ROI of 13.5% of the total tibial length. Analysis of trabecular bone structure was completed using adaptive thresholding (mean of min and max values) in CTan with threshold set at 45–255 for trabecular bone. Cortical analyses were performed 35% of the total tibial length distal from the mineralized zone of the proximal growth plate, and extending for 12% of the total tibial length; the threshold values for cortical bone were set to 79–255 and global thresholding algorithm was used. The 3-dimensional visualization of trabecular and cortical bone was performed with CTVox, where volume-rendered images were pseudo-colored based on grey-scale (pixel) intensity that is reflective of bone mineralization.

### Cell culture

The Kusa4b10, long-bone primary osteoblastic cells and mouse OS cell lines (no authentication performed) were cultured in αMEM (Lonza), 10% non heat inactivated FBS (SAFC Biosciences) and 1% Penicillin/Streptamycin/Glutamine (Life Technologies). OS cell cultures were derived by mincing tumor tissue with a scalpel. The resulting tumor homogenate was transferred to a 6 well plate and allowed to establish in standard culture conditions. Primary cultures were passaged at 60–80% confluence with media changes every 2–3 days. The primary osteoblastic cells were derived from long bone tissue, cleaned and crushed lightly with a mortar-pestle. The suspension was rinsed and diluted with PBS, and this process was repeated until the majority of hematopoietic cells had been removed (solution was clear). The bone fragments were placed in 15ml of collagenase I (3mg/ml) (Worthington), and incubated in a shaking 37°C water bath for 45 minutes. 35ml of PBS + 2% FBS was then added and the cell suspension was sieved. The resulting population of long-bone derived cells was centrifuged at 400g for 5 minutes, the cells were resuspended in culture media and plated onto a 6-well plate. On the next day, the 6-well plate was washed with PBS before adding fresh culture media to remove floating debris. By 48 hours post-derivation, the cells were ready for experiments. For experiments involving *Rosa26*-CreER^T2^ *Recql4* cells, cultures were treated with 500nM Tamoxifen (4-OHT, Merck) to induce Cre activity.

### Cell preparations and flow cytometry

The primary long-bone osteoblastic cells from *Osx*-Cre *Recql4* mice were obtained as described previously [43,53]. Briefly, bone fragments were crushed and colllagenase-digested in a similar process to the derivation of these cells for *in vitro* experimentation. After pelleting the bone derived cells, they were stained with antibodies (Hematopoietic lineage markers, CD31, CD51, Sca1, CD45), and separated on the FACSAria cell sorter (BD Biosciences). Osteoblastic cells (Lineage^-^, CD31^-^, CD51^+^, Sca1^+/-^) were obtained and fractionated into YFP^+^ and YFP^-^ populations.

### Assessment of genomic excision

Tissues from various organs and FACS-sorted osteoblastic cells were subjected to genomic DNA extraction as directed by the manufacturer (Isolate II genomic DNA kit, Bioline). PCR was performed using 40ng of genomic DNA with the sequence-verified primers and conditions (S2 Table). Following that, the PCR products were subjected to electrophoresis on 2.5% agarose gels and visualized on the VersaDoc imaging system (Bio-Rad). The DNA bands were quantitated with the ImageJ software (NIH).

### shRNA

The Recql4 shRNA were in the pLKO.1 vector and were purchased from Sigma-Aldrich (S4 Table). A Luciferase shRNA plasmid was used as a negative control (S4 Table). Ecotropic lentivirus was generated by transient transfection of 293T cells using calcium phosphate and the psPax2 (Addgene plasmid #12260) and pCMV-Eco Envelope plasmid (Cell Biolabs) using standard methods. Kusa4b10 cells were infected by spinoculation in 6-well plates. The cells were passaged onto fresh plates after 48 hours with media containing puromycin (Sigma). The cells were used after 2 days of antibiotic selection when non infected cells had died.

### Apoptosis, cell cycle and senescence analysis

shRNA infected and selected Kusa4b10 cells were harvested and seeded at 100,000 cells per well in a 6 well plate. On the next day the cells were stained with AnnexinV-APC antibody (eBioscience) in 1X binding buffer (0.01M HEPES, pH7.4; 0.14M NaCl; 2.5mM CaCl_2_, eBioscience) for 15 mins at room temperature in the dark. The cells were then washed with 1X binding buffer and 5μg/ml of 7AAD was added to each sample prior to flow cytometry analysis. Both early apoptotic (AnnexinV^+^/7AAD^-^) and late apoptotic (AnnexinV^+^/7AAD^+^) cell populations were added together to determine the total number of apoptotic cells.

For cell cycle analysis the cells were treated with 10μM EdU (Life Technologies) for 1 hour at 37°C. Cells were then harvested, washed in PBS with 1% BSA, and fixed with 2% paraformaldehyde for 15 min at room temperature in the dark. The cells were washed with PBS with 1% BSA and stored as a pellet at 4°C. Cells were processed as described by the manufacturer (EdU labelling kit, Life Technologies) The cells were subjected to flow cytometry to assess the cellular incorporation of EdU.

For cellular senescence 4–5 days post infection/selection, SA-ß-gal staining was performed with Senescence ß-Galactosidase Staining Kit according to the manufacturer’s instructions (Cell Signalling). Briefly, the cells were washed with PBS, then fixed with 2% formaldehyde and 0.2% glutaraldehyde in PBS for 15 min at room temperature. After rinsing the cells with PBS, the fixed cells were incubated with X-gal staining solution and visualised with an inverted microscope.

### *In vitro* differentiation

Osteoblastic cells were seeded and cultured for 3–4 days. After the cells reached confluence, the media was replaced with differentiation media (αMEM, 15% FBS, 50μg/ml L-ascorbic acid, 10mM beta-glycerophosphate) and thereafter changed 2 times per week. Cells were collected on indicated days for analysis.

For mineralization the cells were washed 3 times (1x PBS), fixed with 70% ethanol for 30 mins and stored at 4^°^C in fixative. After washing the cells 3 times in water, they were stained with 0.5% Alizarin Red S (Sigma Aldrich) in water for 30 mins, then 3 washes in water and a 15 min wash in 1x PBS. 1 ml of 10% Hexadecylpyridinium chloride monohydrate (CTP, Sigma Aldrich) in DPBS was added and the plates incubated overnight at room temperature with shaking to elute and quantitate the Alizerin Red S. Absorbance was measured on a PolarStar Plate Reader at OD_562_nm.

### Gene expression analysis

RNA was extracted from OS tissue as previously described [43]. RNA was also extracted from cell lines with the Isolate II RNA kit (Bioline). cDNA was synthesised with the Tetro kit (Bioline) with oligo-(dT) primers. Both SYBR-green and multiplex based probe qPCR were performed on the Stratagene Mx3000P machine (Agilent Technologies) with gene-specific primers (S3 Table). The relative gene expression was normalized to the HPRT housekeeping gene and calculated by the 2^-ΔCT^ method.

### Immunoblot

RIPA buffer extracted protein lysates from shRNA-transduced and selected Kusa4b10 cells were quantified with the Quick-start Bradford assay (Bio-Rad), and separated on a 4–12% Bis-Tris gel in MOPS buffer as directed by the manufacturer (Life Technologies). The gel was then transferred onto a PVDF membrane (Merck-Millipore). The membrane was blocked with 5% milk in TBST and incubated overnight at 4°C with the polyclonal rabbit anti-mouse Recql4 antibody (GL Biochem, 1:500) and the monoclonal anti-mouse α-tubulin antibody (Sigma, 1:5000) as previously described [27]. Subsequently, the blot was probed with horseradish peroxidase-conjugated goat anti-rabbit and goat anti-mouse secondary antibodies respectively (Thermo Scientific, 1:2000). The enhanced chemiluminescence kit (GE Amersham) was applied to the blot, and bands were visualized by exposing the WB to X-ray film.

### Cell preparations and flow cytometry analysis

Peripheral blood was counted on a hematologic analyser (Sysmex KX-21N, Roche Diagnostics). Spleens were weighed and crushed in FACS Buffer (PBS + 2% FBS) to make single-cell suspensions. The bone marrow was recovered by flushing the femurs with FACS Buffer. The bone marrow cell suspensions were then stained with anti-mouse antibodies. Antibodies against murine CD2, CD3e, CD4, CD5, CD8a, CD11b, CD19, CD31, CD34, CD41, CD43, CD44, CD45.1, CD45.2, CD51, B220, CD71, PDGFRα (CD140a), F4/80, Gr1, IgM, Mac1, Sca1, and Ter119 were either biotinylated or conjugated with FITC, phycoerythrin, phycoerythrin-Cy5, peridinin chlorophyll protein-Cy5.5, phycoerythrin-Cy7, allophycocyanin, or allophycocyanin Alexa 750 and were all obtained from eBioscience, Biolegend, BD Biosciences or Life Technologies. Biotinylated antibodies were detected with streptavidin conjugated with Alexa-Fluor 488 (Life Technologies) or Brilliant Violet 605 (eBiosciences) [27,53]. FACS data was collected on an LSRIIFortessa (BD Biosciences) and analyzed with FlowJo software Version 9.0 (Treestar).

### Kaplan-Meier survival analysis and statistical analysis

Mice were monitored daily until they reached humane endpoint criteria. Once the criteria were met, the mice were euthanised. The Kaplan-Meier survival plots, the Log-Rank statistical test and the unpaird 1-tailed Student t-tests with were prepared using Prism 6.0 (Graphpad Software). Census dates are the date of euthanasia of the tumor-bearing animal. All data is presented as mean ± S.E.M. A *P* value <0.05 was considered significant.

## Supporting Information

S1 Fig Histomorphometric analysis of Osx-Cre Recql4 animals. Histomorphometric analysis of the secondary spongiosa of the proximal tibia from *Osx*-Cre *Recql4* ^*+/+*^ and *Recql4* ^*fl/fl*^ mice. (a) Trabecular bone volume (BV/TV), trabecular separation (Tb.Sp), trabecular number (Tb.N), and trabecular thickness (Tb.Th). (b) Osteoid volume per unit bone volume (OV/BV), osteoid surface per unit bone surface (OS/BS), osteoid thickness (O.Th). (c) Number of osteoblasts per unit bone perimeter (N.Ob/B.Pm), osteoclast surface per unit bone surface (Oc.S/BS), and number of osteoclasts per unit bone perimeter (N.Oc/B.Pm). n = 5–7 per genotype; Data presented as mean ± SEM.
(EPS)

S2 Fig Deletion of *Recql4* in osteoblasts does not affect hematopoiesis. (a) Total leukocyte counts, platelet cell counts, red blood counts and hematocrit in peripheral blood of *Osx*-Cre *Recql4* ^*+/+*^ and *Recql4* ^*fl/fl*^ mice. (b) Analysis of hematopoietic cell lineages from the bone marrow of *Osx*-Cre *Recql4* ^*+/+*^ and *Recql4* ^*fl/fl*^ mice. (c) Gross weights and leukocyte counts from the spleens of *Osx*-Cre *Recql4* ^*+/+*^ and *Recql4* ^*fl/fl*^ mice. For (a)-(c): n = 5 per genotype; Data presented as mean ± SEM.
(EPS)

S3 Fig Genomic excision of Recql4 in 4OHT-treated *R26*Cre-ER^T2^ *Recql4* primary osteoblastic cultures. Agarose gel photographs for the assessment of Recql4 genomic excision. 40ng of genomic DNA from long-bone primary osteoblastic cells was obtained from several time-points of 4OHT treatment, and subjected to PCR and gel electrophoresis. The quantification of Recql4 genomic excision was presented in Fig 5B.
(EPS)

S4 Fig Assessing the effects of shRNA-mediated *Recql4* knockdown in Kusa4b10 cells. (a) Position of the various shRNAs that target the mouse Recql4 gene. Arrows in red depict the binding region used for Recql4 qPCR primers. (b) Assessment of Recql4 transcript knockdown in Kusa4b10 cells by qPCR. Relative gene expression levels were normalised to the expression of Hprt. n = 3 biological replicates; Data presented as mean ± SEM. * P<0.05, compared with shRNA-Luciferase, t-test. (c) Assessment of the effects of Recql4 knockdown on p21 and Noxa expression in Kusa4b10 cells. Data are expressed as fold induction relative to transcript expression levels from shRNA-Luciferase cells. n = 3 biological replicates; Data presented as mean ± SEM. * P<0.05, ** P<0.01, compared with shRNA-Luciferase, t-test. (d) shRNA-Luciferase and #5254 cells were cultured in 6-well plates for 3 days post-selection and subjected to senescence-associated B-galactosidase staining. Light microscopic images were taken at 400X magnification. (e) Transcript expression of osteo-lineage genes based on qPCR on shRNA-Luciferase and #5254 cells that were subjected to osteogenic differentiation. The relative gene expression is normalised to the expression of Hprt. Data presented as mean ± SEM from biological replicates.
(EPS)

S5 Fig Histology of primary OS tumours from *Osx*-Cre *p53* ^*fl/fl*^ *Recql4* mice. Paraffin sections were stained for H&E, and light microscopic images were taken at 100X magnification. Scale bar: 200μm.
(TIF)

S1 Table Summary of survival of *Osx*-Cre *Recql4* and osteosarcoma models. (DOCX)

S2 Table Primers used for genomic DNA PCR. (DOCX)

S3 Table Primers used for qRT-PCR. (DOCX)

S4 Table Recql4 shRNA target sequences. (DOCX)
