# Supplementary figures and images for: The DNA Helicase Recql4 Is Required for Normal Osteoblast Expansion and Osteosarcoma Formation
Source: PLoS Genet. 2015 Apr 10;11(4):e1005160. doi: 10.1371/journal.pgen.1005160 (PMC4393104; doi:10.1371/journal.pgen.1005160)

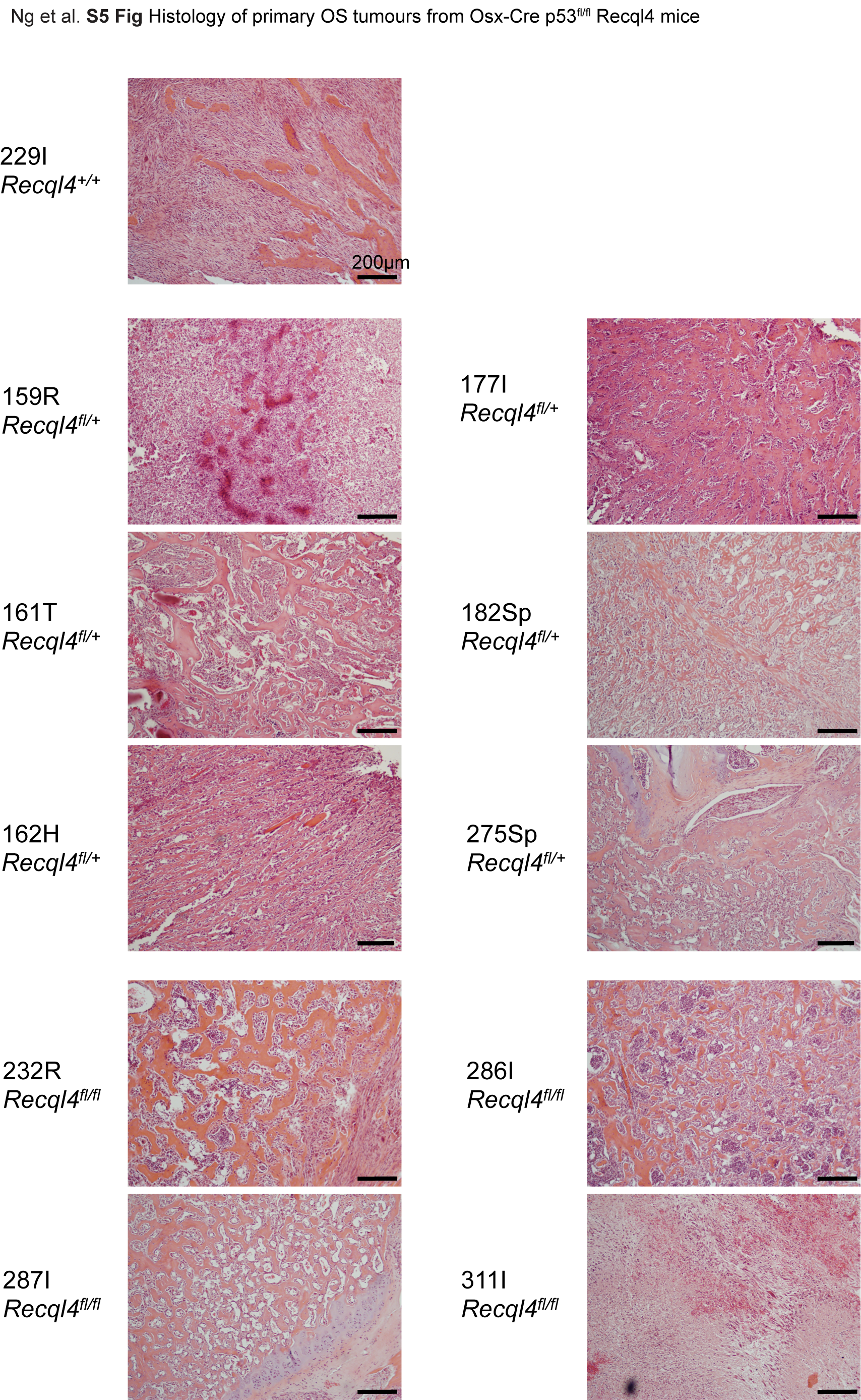

Supplement: S5 Fig — Paraffin sections were stained for H&E, and light microscopic images were taken at 100X magnification. Scale bar: 200μm. (TIF) [file pgen.1005160.s005.tif]
