# Supplementary material for: The DNA Helicase Recql4 Is Required for Normal Osteoblast Expansion and Osteosarcoma Formation
Source: PLoS Genet. 2015 Apr 10;11(4):e1005160. doi: 10.1371/journal.pgen.1005160 (PMC4393104; doi:10.1371/journal.pgen.1005160)
Supplement: S1 Table — (DOCX) [file pgen.1005160.s006.docx]

**Supplemental Table 1** *Summary of Osx-Cre Recql4 models*

| **Model** | | **Sample Number** | **Number of OS tumours seen** | | **Cause of death** | | | | | | | | | | | | | |
| --- | --- | --- | --- | --- | --- | --- | --- | --- | --- | --- | --- | --- | --- | --- | --- | --- | --- | --- |
|  | *Recql4* status |  |  | | Euthanized (700+ day time point) | | | Found Dead | | Lymphoma | | Enlarged/  Distended Stomach | | Malocclusion | | | Other | |
| *Osx*-Cre | *Recql4^+/+^* | N = 6 | 0 | | 3 | | | 0 | | 0 | | 0 | | 0 | | | 0 | |
|  | *Recql4^fl/+^* | N = 5 | 0 | | 4 | | | 0 | | 0 | | 1 | | 0 | | | 0 | |
|  | *Recql4^fl/fl^* | N = 10 | 0 | | 5 | | | 1 | | 1 | | 1 | | 1 | | | 1 | |
| **Model** | | **Median survival in Days (Range)** | | **Distribution of Primary Tumours** | | | | | **Primary Tumour Mutiplicity*** | | **Metastatic Potential** | | **Site of Metastatic Disease** | | | | | |
|  | *Recql4* status |  | | Head | | Long Bones | Others (Axial) | |  | |  | | Lung | | Liver | Kidney | | Other |
| *Osx*-Cre *p53^fl/fl^* | *Recql4^+/+^* | N = 3 | 247  (177-432) | 0 | | 2 | 2 | | 1/3  (33.3%) | | 1/3  (33.3%) | | 1 | | 0 | 0 | | 0 |
|  | *Recql4^fl/+^* | N = 14 | 245.5  (173-387) | 1 | | 6 | 8 | | 3/14  (21.4%) | | 8/14  (57.1%) | | 7 | | 1 | 0 | | 0 |
|  | *Recql4^fl/fl^* | N = 12 | 346  (240-508) | 1 | | 5 | 13 | | 5/12  (41.7%) | | 3/12  (25.0%) | | 3 | | 0 | 0 | | 0 |
