# Supplementary material for: The DNA Helicase Recql4 Is Required for Normal Osteoblast Expansion and Osteosarcoma Formation
Source: PLoS Genet. 2015 Apr 10;11(4):e1005160. doi: 10.1371/journal.pgen.1005160 (PMC4393104; doi:10.1371/journal.pgen.1005160)
Supplement: S2 Table — (DOCX) [file pgen.1005160.s007.docx]

**Supplemental Table 2** *Primers used for genomic DNA PCR*

| Allele | Primer Sequence 5’ – 3’ | Number of Cycles; Annealing Temperature | Expected band sizes |
| --- | --- | --- | --- |
| p53 | F - cacaaaaacaggttaaacccag  R - agcacataggaggcagagac | 30 cycles; 58ºC | Wt: 288bp  Flox: 370bp |
| Recql4 | F - cactctagaagagggagtcagatgg  Ex - cgcgcgaaagctgaggagtt  R - acagcaacagaacagcaactacg | 30 cycles; 60ºC | Wt: 165bp  Excised: 256bp  Flox: 325bp |
