# Supplementary material for: The DNA Helicase Recql4 Is Required for Normal Osteoblast Expansion and Osteosarcoma Formation
Source: PLoS Genet. 2015 Apr 10;11(4):e1005160. doi: 10.1371/journal.pgen.1005160 (PMC4393104; doi:10.1371/journal.pgen.1005160)
Supplement: S3 Table — (DOCX) [file pgen.1005160.s008.docx]

**Supplemental Table 3** *Primers used for qRT-PCR*

Multiplex qPCR forward (F) and reverse (R) primer and probe sequences

| Gene | Primer Sequence 5’ – 3’ | Probe Sequence 5’ – 3’ | 5' Fluorophore &  3' quencher |
| --- | --- | --- | --- |
| Hprt1 | F - ccccaaaatggttaaggttgc  R - aacaaagtctggcctgtatcc | cttgctggtgaaaaggacctctcgaa | HEX  Iowa Black FQ |
| Runx2 | F - ctattaaagtgacagtggacgg  R - gcgatcagagaacaaactagg | cgggaaccaagaaggcacagaca | FAM  Iowa Black FQ |
| Osx | F - cctctcccttctccctctc  R - ctggagccatagtgagcttc | tcctcggttctctccatctgcct | Cy5  Iowa Black RQ-Sp |
| Alk Phos | F - actgatgtggaatacgaactgg  R - agttcagtgcggttccag | agtgggaatgcttgtgtctgggt | ROX  Iowa Black RQ-Sp |
| Pthr1 | F - ccccgagtctaaagagaacaag  R - gtaatcgggacaaggtactgc | agtgggacaacatcgtttgctgg | ROX  Iowa Black RQ-Sp |
| Ocn | F - accatctttctgctcactctg  R - gttcactaccttattgccctcc | acctcacagatgccaagccca | FAM  Iowa Black FQ |
| Sost | F - acaaccagaccatgaaccg  R - caggaagcgggtgtagtg | acgccaaagatgtgtccgagtacag | Cy5  Iowa Black RQ-Sp |
| p53 | F - ccccactttcttgaccattg  R - atgttccgggagctgaatg | agttaaaggatgcccatgctacagagg | FAM  Iowa Black FQ |
| Noxa | F - acactcgtccttcaagtctg  R - ccggacataactgtggttct | tcatcctgctcttttgcgacttccc | FAM  Iowa Black FQ |
| p21 | F - caatctgcgcttggagtga  R - cttgtcgctgtcttgcact | agaaatctgtcaggctggtctgcc | FAM  Iowa Black FQ |

Sybr-green qPCR primer sequences

| Gene | Forward (F) Primer Sequence 5’ – 3’ | Reverse (R) Primer Sequence 5’ – 3’ |
| --- | --- | --- |
| Hprt1 | tgattagcgatgatgaaccag | agagggccacaatgtgatg |
| Sost | gacacatctttggcgtcatag | ccacagaggtcatccca |
| Dmp1 | ccagagggacaggcaaatag | ctggactgtgtggtgtctgc |
| Bsp | ccgaagcctatgggaccac | ataagctcggtaagtgtcgcc |
| Recql4 | ctctaccgtgagtaccgtaacctaa | agtaggctctgttttggcatagact |
