# Supplementary material for: The DNA Helicase Recql4 Is Required for Normal Osteoblast Expansion and Osteosarcoma Formation
Source: PLoS Genet. 2015 Apr 10;11(4):e1005160. doi: 10.1371/journal.pgen.1005160 (PMC4393104; doi:10.1371/journal.pgen.1005160)
Supplement: S4 Table — (DOCX) [file pgen.1005160.s009.docx]

**Supplemental Table 4** *shRNA Lentiviral plasmids and their target sequences*

| Target Gene | Clone Name | Identifier # | Target sequence (5’ 🡪 3’) |
| --- | --- | --- | --- |
| Recql4 | TRCN0000375330 | #5330 | gagactctgctgtgctatttg |
|  | TRCN0000366557 | #6557 | ctagacagagggaactatatt |
|  | TRCN0000115254 | #5254 | cctagacagagggaactatat |
|  | TRCN0000115255 | #5255 | cctggattcagttatcattta |
|  | TRCN0000379295 | #9295 | ttggcatagctggcgagtttg |
| Luciferase | TRCN0000072259 | Luciferase | cgctgagtacttcgaaatgtc |
